# Intraoral Superficial Angiomyxoma of the Upper Alveolus: Report of a Unique Case

**DOI:** 10.1155/2012/859021

**Published:** 2012-08-05

**Authors:** Ravindra S. V., M. Srinivasa Raju, Sunitha J. D., Neeraj Taneja, Sunira Chandra, Shveta Mahajan, Eesha Panwar

**Affiliations:** ^1^Department of Oral Medicine and Radiology, Teerthanker Mahaveer Institute of Dental Sciences and Research Centre, Teerthanker Mahaveer University, Bagarpur, Delhi Road, Moradabad 244001, India; ^2^Department of Oral Pathology and Microbiology, Teerthanker Mahaveer Institute of Dental Sciences and Research Centre, Teerthanker Mahaveer University, Bagarpur, Delhi Road, Moradabad 244001, India

## Abstract

Angiomyxomas are relatively a group of uncommon myxoid mesenchymal tumors characterized by frequent local recurrences and show lack of malignant potential. Basically three types of angiomyxomas are recognized superficial, aggressive, and angiomyofibroblastoma. Though the angiomyxomas are rarely reported in the head and neck region, the paper shows reported cases intraorally in the buccal mucosa and floor of the mouth. Here, the authors report a rare case of angiomyxoma presenting as a growth in the upper posterior alveolar mucosa.

## 1. Introduction

Angiomyxomas are a group of relatively uncommon myxoid mesenchymal tumors characterized by frequent local recurrences and lack of metastatic potential. Three types of angiomyxomas are recognized: aggressive, superficial and angiomyofibroblastoma [1]. Superficial angiomyxoma, also known as cutaneous myxoma [2], was first described by Allen et al. in 1988 and in greater detail in 2000 [3]. Although there have been many reported cases in the head and neck, including sites such as the chin, lip, cheek, nose, ear, forehead, face, and neck; truly intraoral cases are extremely rare [2] with 4 previously reported cases till date occurring in the buccal mucosa and floor of the mouth [2–5]. To the best of our knowledge, we report the first case of an intraoral superficial angiomyxoma, occurring in the upper alveolar mucosa.

## 2. Case Report

**Figure 1:**
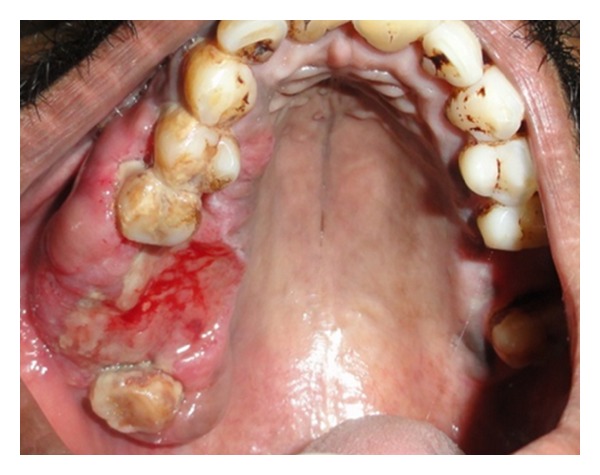
Diffuse, reddish, and slightly ulcerated lesion in the right alveolar region.

**Figure 2:**
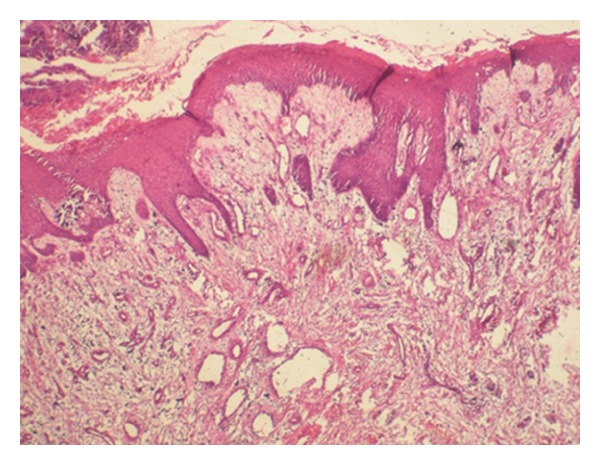
Photomicrograph showing stratified squamous epithelium overlying myxoid connective stroma with prominent vasculature (hematoxylin and eosin stain, 4x).

**Figure 3:**
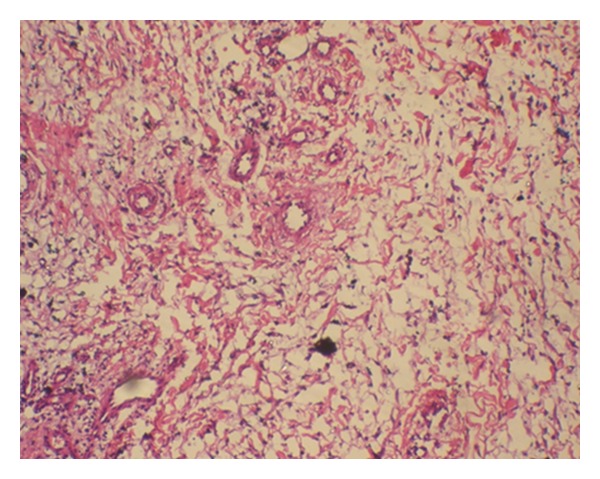
Photomicrograph showing loose collagenous myxomatous stroma permeated by spindle shaped cells and nonarborizing blood vessels (hematoxylin and eosin stain, 10x).

**Figure 4:**
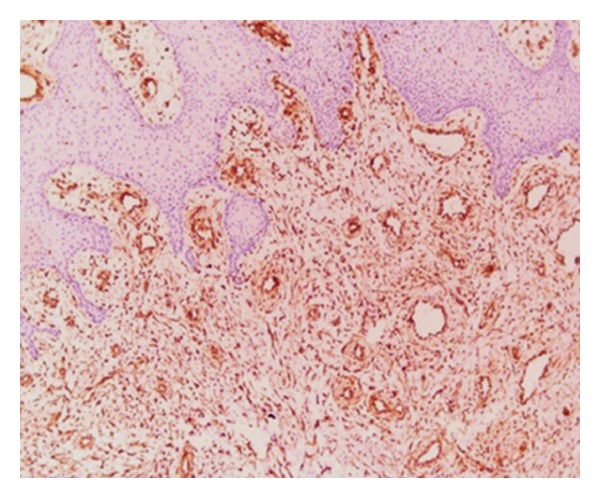
Many tumor cells stained strongly for vimentin (10x).

**Figure 5:**
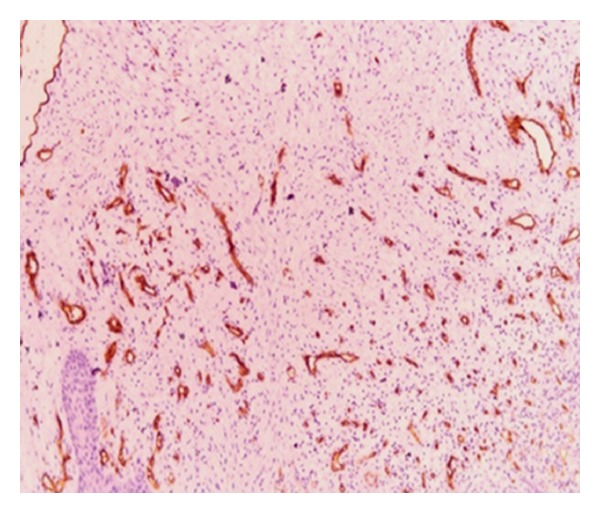
The endothelial cells of the blood vessels stained strongly for CD34 (10x).

A 30-year-old male patient presented with a slow-growing, soft mass in the right upper posterior alveolar region, present since 2 years and which was associated with mild pain upon eating (Figure 1). Patient had noticed a mobile tooth in the same region about a year and a half back and had got it extracted. The swelling had continued to grow slowly after the extraction. His medical history was noncontributory. Examination showed that the lesion was diffuse, reddish, slightly ulcerated, and measuring about 3 × 3 cm in size. It was rubbery to firm in consistency and mildly tender, with slight bleeding on palpation. Radiographs were taken but no changes were observed. Incisional biopsy was performed. Microscopic examination revealed epithelium overlying loose myxoid stroma accompanied by a prominent vasculature (Figure 2). Myxoid stroma showed a scattered spindle to stellate-shaped cells which had distinct borders and oval nuclei. There was no cellular or nuclear atypia or hyperchromasia and mitotic activity and necrosis were not present. Small, thin-walled curvilinear blood vessels were prominent throughout the stroma. A mild inflammatory infiltrate was present predominantly neutrophils (Figure 3). An immunohistochemical staining was performed using vimentin and CD34 antigens. Most of the stromal tumor cells were immunopositive for vimentin (Figure 4) and the endothelial cells of the blood vessels displayed immunoreactivity for CD34 (Figure 5). This confirmed the diagnosis of superficial angiomyxomas. Treatment was not done due to patient unwillingness.

## 3. Discussion

Superficial angiomyxomas are rare distinctive, benign, cutaneous soft tissue lesions with a predilection for the trunk, head, and neck; other sites being lower extremities and genital area [2]. Cutaneous superficial angiomyxomas typically present as polypoid or papulonodular lesions that may be confused with a cyst, skin tag, or neurofibroma. There is a slight male predilection and most cases present in middle age although rare congenital examples have been reported as well [3]. However, these tumors can also occur within the oral cavity [2]. A brief outline of the previously reported oral superficial angiomyxomas is summarized in Table 1. There are still too few documented intraoral cases to draw meaningful clinical comparisons. The distinctive histological features of superficial angiomyxoma include a multilobular growth pattern composed of spindle-shaped to stellate cells in a copious myxoid stroma. Small, thin-walled vessels are prominent and there is presence of stromal inflammatory cells, especially neutrophils which is an important diagnostic clue [1, 2].

The main histologic differential diagnosis for intraoral tumors includes aggressive angiomyxoma, soft tissue myxoma, angiomyolipoma, myxoid nerve sheath tumor (neurothekeoma), myxoid neurofibroma, oral focal mucinosis, and myxofibroma or odontogenic myxoma.

Aggressive angiomyxoma can be distinguished from its superficial and angiomyofibroblastoma counterparts by the proliferation of spindle- or satellite-shaped cells that are widely separated by loose myxoid stroma in which there are prominent and large vascular components [1, 2].

Angiomyolipoma is composed of a mixture of thick-walled blood vessels, smooth muscle, and adipose tissue found mostly in the kidney [4]. Neither smooth muscle nor adipose tissue were seen in the tumor tissue of the current case. Nerve sheath myxoma has smaller individual nodules, is less vascular with cells arranged concentrically like a Pacinian corpuscle, and contains occasional eosinophilic histiocytic cells. The cells of myxoid neurofibroma are typically slender with wavy nuclei and intralesional nerve bundles. Oral focal mucinosis is typically acellular with very few blood vessels, lack of a lobular architectural pattern, and no stromal inflammation [2]. Myxofibromas or odontogenic myxomas are central lesions that are diffuse and nonlobulated, with no stromal inflammation, and may contain odontogenic epithelial rests. Superficial angiomyxomas are generally immunoreactive with vimentin and CD34, which was consistent with the present case. Treatment is by localized surgical excision, with careful followup owing to its high rate of local recurrence between 20% and 40% [2–4]. Superficial angiomyxomas have an overall good prognosis as this lesion stays superficial, without affecting deeper structures [2, 4].

## 4. Conclusion

Superficial angiomyxoma is clearly a very rare neoplasm of the oral cavity and should be included in the differential diagnosis of myxoid intraoral soft tissue neoplasms.

## Figures and Tables

**Table 1:** Reported cases of intraoral superficial angiomyxoma.

Reference	Site	Age	Gender	Race	Size (mm)	Presentation	Clinical impression
Chen et al. [4]	Right buccal mucosa	19 yrs	Male	Chinese	50 × 35 × 30	Slow growing painless lump present for 2 years	Soft tissue tumor

Gardner [3]	Floor of the mouth	69 yrs	Female	European	10 × 12 × 12	Slow growing painless lump present for 3 years	Lipoma

Meer and Beavon [2]	Right buccal mucosa	37 yrs	Female	African	45 × 32 × 20	Slow growing painless lump present for 2 years	Lipoma

Mokhtar et al. [5]	Floor of the mouth	6 months	Male	Malaysian	50 × 36 × 26	Slow growing swelling noticed when patient was 5-month-old	Soft tissue tumor/cystic swelling

Present case	Upper posterior alveolar region	30 yrs	Male	Indian	30 × 30	Slow growing painless swelling present for 1 and a half years	Soft tissue tumor
